# Acute Toxicity Assessment of Orally Administered Microplastic Particles in Adult Male Wistar Rats

**DOI:** 10.3390/toxics12030167

**Published:** 2024-02-22

**Authors:** Ivana Guševac Stojanović, Dunja Drakulić, Ana Todorović, Jelena Martinović, Nenad Filipović, Zoran Stojanović

**Affiliations:** 1 Department of Molecular Biology and Endocrinology, VINČA Institute of Nuclear Sciences-National Institute of the Republic of Serbia, University of Belgrade, Mike Petrovića Alasa 12-14, 11351 Belgrade, Serbia; drakulic@vin.bg.ac.rs (D.D.); anato@vin.bg.ac.rs (A.T.); jelenazlatkovic@vin.bg.ac.rs (J.M.); 2 Group for Biomaterials and Biomedical Applications, Institute of Technical Sciences of SASA, Knez Mihailova Street 35/4, 11000 Belgrade, Serbia; nenad.filipovic@itn.sanu.ac.rs

**Keywords:** polyethylene terephthalate (PET), single treatment, general health status, serum biomarkers

## Abstract

While the effects of chronic exposure to microplastic particles (MPs) are extensively studied, the outcomes of a single treatment have received relatively less attention. To investigate MPs’ potential acute toxicity, including their impact on general health status (victual consumption, sensorimotor deficits, and clinical toxicity signs) and serum biochemical parameters (markers of organ/tissue function and oxidative stress indicators), we administered thoroughly characterized MPs (1.4, 35, or 125 mg/kg), generated from polyethylene terephthalate (PET) bottles, to adult male Wistar rats via oral gavage. The MPs’ short-term effects were assessed with well-established tests and methods. The results point to the absence of sensorimotor deficits and clinical toxicity signs, while levels of markers of liver, heart, and kidney function were altered in all MP groups. Decreased victual consumption and increased levels of oxidative stress indicators were evident following treatment with the two higher MP doses. Presented data indicate that examined MPs are able to initiate the development of local changes in tissues and organs within a short time frame, potentially leading to their damage and dysfunction. This study may increase the awareness of the detrimental effects of plastic contamination, as even a single exposure to MPs may provoke adverse health outcomes.

## 1. Introduction

Plastic has become an indispensable and versatile material used in the production of various items with a wide range of application in everyday life [1]. At least 45 different types of plastic materials have been developed to satisfy market demands, among which polyethylene terephthalate (PET) stands as one of the most commonly used [2]. The utilization of PET and other plastic materials increases simultaneously with the growth of the human population and yields problems in their disposal. Moreover, in recent years, the presence of plastic particles, particularly those with a diameter of less than 5 mm, generally termed microplastic particles (MPs), has emerged as a new global environmental threat and hazard [3]. MPs, specifically those smaller in size, are widely used in cosmetic products, toothpastes, sun creams, and other merchandise. Found ubiquitously across the planet, MPs are absorbed by plants and ingested by other organisms residing in contaminated areas, eventually making their way into the human body via the food chain [4]. Furthermore, MPs that are unintentionally released during food and beverage production, packaging processes, and transportation [4] through victuals additionally enter the human body. Reports have indicated the presence of MPs, ranging up to 150 µm in size, in lymph, portal vein, blood, feces, and other bodily components. In some instances, they may infiltrate into organs, leading to systemic vulnerability [5,6,7]. However, it should be noted that MPs > 150 µm are believed to have limited absorption potential, and they create only local effects on the immune system and inflammation within the gastrointestinal tract (GIT) [7].

Until recently, the amount of MPs consumed by humans was unknown [8]. Consequently, experimental studies involving MP utilization often lacked realism, as they exploited MPs in higher magnitudes than are detected in the environment [9]. However, recently, Senathirajah and colleagues [8] estimated that globally, average MP ingestion per week ranges from 0.1 to 5 g depending on a combination of many factors, for example, the characteristics of MPs as well as demographic characteristics.

Due to the scarcity of data in the literature regarding the impact of a single instance of MP exposure in mammals, we aimed to investigate the potential acute toxicity in adult male Wistar rats provoked by MPs generated by filing PET bottles of a worldwide well-known soft drink. This study exploited MPs (with a median diameter of 85 µm), and administered them in three doses (1.4, 35, 125 mg/kg), representing possible lower, middle, and higher daily amounts of ingested MPs after a short period of 24 h following the treatments.

## 2. Materials and Methods

### 2.1. Microplastic Particle Preparation and Physicochemical Characterization

#### 2.1.1. Microplastic Particle Preparation

The PET bottles of a worldwide well-known soft drink brand were purchased from local stores. The bottles were thoroughly washed with distilled water (dH_2_O), rinsed with Milli-Q water, and allowed to completely dry. To make them brittle for easier manipulation, bottles underwent three cycles of cooling to −50 °C and subsequent heating to 40 °C. Then, the bottles were filed. The produced sawdust-like MPs were collected and run through a laboratory sieve with a mesh size of 160 µm.

#### 2.1.2. Microplastic Particle Physicochemical Characterization

The OPTICA B-500MET light microscope (Optica SRL, Ponteranica, Italy) was used to observe MPs after dispersing them in ethanol, depositing them on slides, drying them, and exposing them to transmitted polarized/unpolarized light. The morphology of the particles was presented using optical microscope images.

The qualitative analysis was performed using Fourier-transform infrared (FTIR) spectroscopy. FTIR spectra were recorded in the range of 400–4000 cm^−1^ using a Thermo Scientific Nicolet iS10 Spectrometer equipped with a Smart iTX accessory (Thermo Scientific, Inc., Waltham, MA, USA) at 4 cm^−1^ spectral resolution and with 32 scans. FTIR spectra were collected in the reflection mode with the built-in diamond attenuated total reflectance (ATR) sampling technique. The OMNIC Software 9.7.46 (Thermo Scientific, Inc., Waltham, MA, USA) was used for the acquisition, processing, analysis, and management of FTIR data in a graphical environment. The results are presented as a transmission spectrum.

The MPs’ size distribution was determined after the sonication of suspended powders in 96% ethanol for 60 s using the laser diffraction (LD) particle size analysis method (Malvern Instruments Mastersizer 2000, Malvern, UK), and the obtained results are presented as a frequency curve.

### 2.2. Acute Oral Toxicity Study

#### 2.2.1. Animals and Treatments

For the experiment, the proposed weekly low (0.1 g), middle (2.5 g) [8], and high doses (8.5 g) [10] were divided by an average body mass (60 kg) [11] and subsequently divided by 7 (for the days in a week) to estimate the daily ingested dose for humans. The acquired number represents the human-equivalent dose (HED).

The animal-equivalent doses of daily MP intake were calculated using the following formula:

AED (mg/kg) = HED (mg/kg) × Km ratio

where AED represents the animal-equivalent dose and the Km ratio is the correction factor estimated by dividing the average body mass (kg) of the species by their body surface area (m^2^) (6.2 for rats) [12]. Thus, the calculated AEDs are 1.4 mg/kg, 35 mg/kg, and 125 mg/kg, and these doses are further employed in the study.

Research procedures performed on animals were approved by the Ethical Committee for the Use of Laboratory Animals of VINČA Institute of Nuclear Sciences–National Institute of the Republic of Serbia, University of Belgrade, Republic of Serbia (protocol authorization number 323-07-03460/2021-05) and were in accordance with the European Communities Council Directive (2010/63/EU for animal experiments).

The experiments were performed on young adult male Wistar rats (n = 25) obtained from the local colony and separate litters. Animals were housed (2–3 per cage) under the standard conditions of 12 h light/dark regime, constant ambient temperature (22 ± 2 °C), and ad libitum access to food and water.

Animals were randomly divided into 5 groups: intact animals (I); rats that received 2.5 mL of Milli-Q (Q); rats that received MPs dispersed in 2.5 mL of Milli-Q at doses of 1.4 mg/kg (P1), 35 mg/kg (P2), or 125 mg/kg (P3). Intragastric administration of MPs was performed using a reusable stainless steel feeding needle (16–G4”, 3 mm ball diameter, Cadence Inc., Staunton, VA, USA).

#### 2.2.2. General Health Status

##### Victual Consumption Estimation

The rats from all experimental groups, except group I, fasted for 4 h before the treatments were applied, while pre-weighed food and water (in g and mL) were returned 2 h after the treatments. The consumed food and water were estimated 24 h after the treatments by subtracting the weight of leftovers from initially provided victuals. The results are presented as the mean of the values of the group ± SEM.

##### Neurological Testing

A 6-point Garcia neurological test for evaluating various sensorimotor deficits (https://med.stanford.edu/sbfnl/services/bm/sm/neurological.html, accessed on 14 February 2024) was conducted 24 h before and after the treatments. The following parameters were examined by the researcher, who was blinded to the experimental setup: spontaneous activity, forepaw outstretching, climbing, symmetry in four limb movements, body proprioception, and response to vibrissae touch. All investigated parameters were rated with 0, 1, 2, or 3 points. The total score was expressed as the sum of average scores of all investigated parameters and was graded on a scale from 0 to 18 (0 being minimum and 18 representing maximum) [13]. The results are presented as the mean of the values of the group ± SEM.

##### Analysis of Clinical Toxicity Signs

The clinical signs of toxicity, including agitation, convulsions, ataxia, touch response, piloerection, sleepiness, lethargy, respiratory distress, and mortality were carefully monitored throughout the study. These observations were made at various time intervals, including before and after fasting, within the first 30 min after the treatments, three times within 3–4 h after the treatments, and twice the following day, considering that the final assessment was 24 h after the treatments (before sacrificing the animals) [14]. The results are presented descriptively, utilizing a scale to indicate the severity of the observed effects. The scale includes symbols such as − (for no effect), + (for mild effect), ++ (for moderate effect), and +++ (for major effect).

#### 2.2.3. Biochemical Parameters in Serum

The animals were decapitated 24 h after the treatments using Harvard Apparatus guillotine (Holliston, MA, USA). Following decapitation, blood samples were collected and subjected to centrifugation at 3000 rpm for 15 min utilizing Heraeus Labofuge GL (Heraeus, Hanau, Germany). The resulting serum was then stored at −80 °C until further processing.

##### Common Health Biochemical Marker Assessment

A biochemical blood analyzer, COBAS Integra 400 plus analyzer (Roche, Rotkreuz, Switzerland), was used to determine the levels of aspartate transaminase (AST (U/L)), alanine transaminase (ALT (U/L)), bilirubin (mmol/L), gamma-glutamyl transferase (GGT (U/L)), alkaline phosphatase (ALP (U/L)), cholesterol (mmol/L), high-density lipoprotein (HDL (mmol/L)), low-density lipoprotein (LDL (mmol/L)), triglycerides (mmol/L), glucose (mmol/L), urea (mmol/L), and creatinine (µmol/L) in serum. The results are presented as the mean of the values of the group ± SEM.

##### Oxidative Capacity Estimation

Serum oxidative stress indicators prooxidant–antioxidant balance (PAB), advanced oxidation protein products (AOPP), and end products of lipid peroxidation–malondialdehyde (MDA) and 4-hydroxinonenal (HNE) were estimated following the protocols described in the study by Miletic and colleagues [15]. Specifically, for PAB assessment, a 96-well microtiter plate was utilized, whereby 200 µL of a working solution consisting of 1 mL tetramethylbenzidine (TMB) cation solution with 10 mL TMB solution was added and mixed with 10 µL of sample or standard (various ratios of hydrogen peroxide and uric acid mixture). After the incubation in a dark environment for 12 min at 37 °C, the reaction was halted by adding 100 µL of 2 M HCl, and absorbance levels were read at 450 nm using the microplate reader (Tecan Sunrise, Tecan Group Ltd., Männedorf, Switzerland). PAB values in the samples were expressed in arbitrary units (HK) determined by calculating the percentage of H_2_O_2_ present in the standard solution. To determine concentrations of AOPP, 20 µL of the sample was mixed with 5 µL of potassium iodide, 80 µL of phosphate-buffered saline (pH 7.4), and 10 µL of acetic acid. The resulting reaction mixture was subjected to absorbance measurement at 340 nm using a previously defined microplate reader, and the AOPP level was expressed in µmol/L of chloramine-T equivalents. Finally, levels of MDA and HNE were estimated in the reaction mixture of the sample and working solution, while hydrochloric acid was added to assess the MDA level, and methane sulfonic acid was added to evaluate the level of HNE. The absorbance of the obtained supernatants was measured at 580 nm after the incubation and centrifugation of the reaction mixture. The levels of MDA and HNE were calculated using the corresponding standard curves and reported in µM. The results of the oxidative stress indicators are presented as a percentage of the mean of the values in Q ± SEM.

### 2.3. Data Analysis

For the analysis of the obtained data, the GraphPad Prism 8.02 software package (Boston, MA, USA) was used. The normality of the data was examined using the Shapiro–Wilk test, and for the data that followed a Gaussian distribution, the one-way analysis of variance (ANOVA) was used to assess the differences between the means of the groups, followed by Tukey’s post hoc test. Data that were not normally distributed were analyzed using the Kruskal–Wallis test followed by Dunn’s multiple comparison test. The values of *p* < 0.05 were considered statistically significant.

## 3. Results and Discussion

The PET is a semicrystalline, colorless, hygroscopic resin produced through the esterification of terephthalic acid with ethylene glycol [16]. This type of plastic occupies a pivotal place among various types of plastics, due to its extensive utilization in textile and packaging industries, including food, water, and soft drink packaging [17]. Only 28.4% of the total PET production is recycled into fibers, sheets, films, and bottles, leaving the majority of PET waste disposed into the environment [17], with undefined impacts on humans, animals, and plants. There is compelling evidence that even ordinary activities in our daily routines, such as tearing plastic bags or twisting/opening bottle caps, can result in the generation of MPs in consumer products, primarily in the form of fragments and fibers [18].

**Figure 1:**
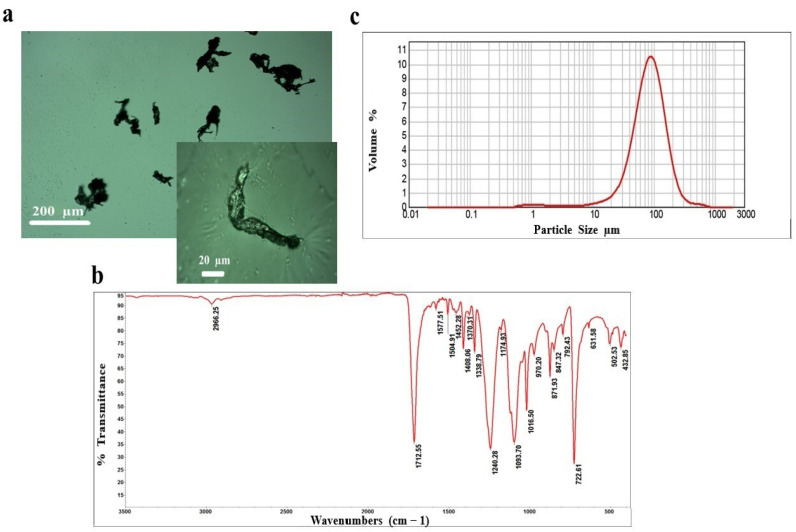
Physicochemical characteristics of microplastic particles (MPs) obtained by filing polyethylene terephthalate (PET) bottles. (**a**) Images of generated, irregularly shaped MPs with higher aspect ratios obtained by optical microscopy; (**b**) Fourier-transform infrared (FTIR) spectra validated that the chemical composition of particles is PET; (**c**) laser diffraction (LD) spectra provided insight into the size distribution of MPs, with a median diameter of 85 µm.

The uniform spherical microbeads purchased from scientific suppliers are mostly employed in animal toxicity studies, as well as the pre-produced pellets obtained from plastic manufacturers and fibrous MPs prepared using cryogenic methods, or irregularly shaped plastic particles (200 µm–5 mm) [9]. To our knowledge, our study represents the first animal toxicity assessment that exploited a novel methodology for obtaining MPs generated from PET. By filing the bottles, in a cost-effective, simple, and rapid in-house manner, the generated particles with broader size distribution were obtained, and their application outcomes in animals more accurately reflect the effects of exposure to MPs in the environment. According to the images acquired by the optical microscope, MPs exhibit an irregular shape with higher aspect ratios (Figure 1a), being similar to those atypical and variously shaped MPs detected in the environment [19].

The FTIR analysis confirmed their PET origin and indicated that the MP samples exhibit several peaks specific to polyesters (Figure 1b). The most dominant and significant peaks are assigned to the following bands: carbonyl C=O at 1712 cm^−1^, aromatic C=C in the region 1615–1453 cm^−1^, bending C–H at 1408 and 1339 cm^−1^, stretching C–C–O at 1240 cm^−1^, stretching C–O at 1094 cm^−1^, bending aromatic C–H at 1016 cm^−1^, and wagging C–H in the aromatic ring at 722 cm^−1^, with the less intensive one at 792 cm^−1^, which is in accordance with previously published data [20,21]. Moreover, LD analysis confirmed their smaller size (d10 = 35 µm and d90 = 170 µm) with a median diameter of 85 µm (Figure 1c).

Upon MP characterization, adult male Wistar rats were exposed to a single MP dose and their general health status, along with biochemical parameters in serum, were evaluated as previously outlined. The in vivo study revealed no differences in all investigated parameters between intact animals and Milli-Q-treated animals (Supplementary Material, Tables S1–S5); therefore, the rats in the Q group served as controls. According to the obtained results, the treatments provoked significant changes in consumption of food (F_(3, 16)_ = 4.494, *p* = 0.0181) and water (F_(3, 16)_ = 6.162, *p* = 0.0055), and decreases were observed in the P2 and P3 groups compared to the Q group (*p* = 0.0379 and 0.0460 for food intake; *p* = 0.0125 and 0.0310 for water intake, respectively (Table 1). These findings suggest that the two higher MP doses adversely impacted the animals, resulting in dysregulation of their appetite and thirst, which might be used as a potential indicator of treatment toxicity.

**Figure 2:**
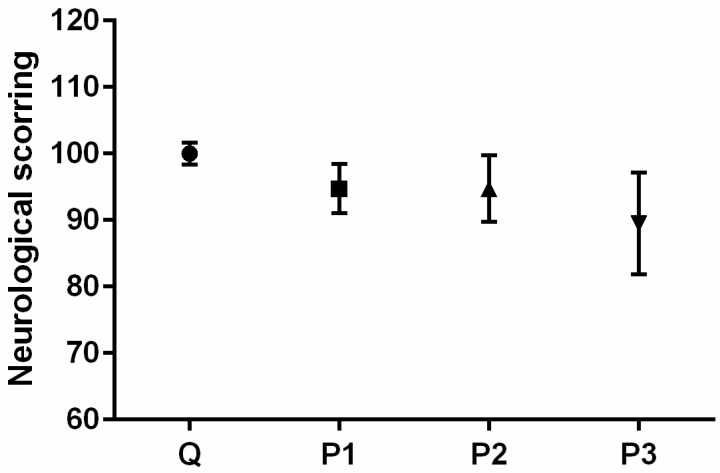
Sensorimotor functions as indicators of the general health status of adult male Wistar rats 24 h after intragastric treatments. Q group—rats that received 2.5 mL of Milli-Q; P1 group—rats that received 1.4 mg/kg of microplastic particles (MPs) dispersed in 2.5 mL of Milli-Q; P2 group—rats that received 35 mg/kg of MPs dispersed in 2.5 mL of Milli-Q; P3 group—rats that received 125 mg/kg of MPs dispersed in 2.5 mL of Milli-Q. Data are presented as the mean ± SEM, whereas the values of the Q group were set as 100%.

This could imply that a certain amount of MPs might be retained within the digestive tract, potentially leading to a false sense of satiety in rats. Critchell and Hoogenboom [22] indicated that although there is some evidence supporting the notion that chronic ingestion of plastic might cause abrasions and lesions within the GIT, the MPs found in the stomach or GIT of some animals induced no obvious harm. The ingested MPs may be retained in the digestive tract and also egested by feces, or they may be uptaken by the epithelial lining of the gut via phagocytosis [23]. In the latest case, subsequent systemic distribution is then possible [23,24], as well as their translocation from the gut cavity to various organs, including the testicles, liver, blood, and, strikingly, to the brain [10,23,25], potentially causing behavioral disruptions, including anxiogenic effects, depression-like behaviors, and altered cognitive functions [23]. However, Rafiee and colleagues [23] demonstrated that chronic exposure to polystyrene nanoparticles in rats had no significant impact on all neurobehavioral tests, but some signs of toxicity were observed, such as decreased locomotor activity. In our study, we sought to investigate neurological and clinical deficiencies following a single MP treatment. It should be noted that none of the animals exhibited sensorimotor or clinical deficits before the experiment. Although comparing to Q group, results of neurological testing revealed a very subtle decrement in sensorimotor functions in all MP-treated groups (Figure 2), these changes were not sufficient enough to reach statistical significance, indicating preserved sensorimotor functions.

Clinical signs of toxicity were also absent in all investigated groups (Table 2). Our findings might be attributed to acute exposure of rats to MPs and the absence of a cumulative effect that could occur with prolonged MP intake, as previously reported [23,26].

The data in the literature emphasize that exposure to various toxic compounds might induce changes in highly vascularized organs, such as the liver, heart, and kidneys, which are reflected by changes in serum biochemical parameters. The toxicity of these compounds might be assessed by monitoring changes in serum biochemical parameters, which serve as indicators of certain organ functions [14,27]. For instance, liver injury might lead to abnormal levels of aminotransferases in serum, such as AST and ALT, which catalyze the transfer of the α-amino group of aspartate or alanine to the α-keto group of ketoglutarate using pyridoxal phosphate (vitamin B6) as a cofactor [28,29]. Both of them are widely distributed throughout the body, with liver being the predominant organ for the accumulation. While AST is also diffusely present in heart, skeletal muscle, kidneys, brain, and red blood cells, ALT is found in lower concentrations in skeletal muscle and kidneys [30,31]. As shown in Table 3, the treatment affected the level of AST (F_(3, 16)_ = 5.366, *p* = 0.0095), which was elevated in the P2 and P3 groups compared to the Q group (*p* = 0.0351 and 0.0190 respectively), suggesting that even a single dose of MPs after a short period could potentially trigger alterations in organ function as a result of inflammation and/or damage of liver cells.

These observations warrant further investigation and study. Interestingly, the treatment affected ALT level (F_(3, 16)_ = 4.271, *p* = 0.0215), and the decrease in ALT level was observed in the P1 group with respect to the Q group (*p* = 0.0153) (Table 3). Low levels of ALT can indicate a vitamin B6 deficiency, which can be associated with liver or kidney conditions, or inflammatory diseases [32,33,34,35]. There are suggestions that lower serum ALT concentrations might occur as a consequence of the decreased hepatocellular function or modulations in enzyme release and/or activity and can be interpreted as damage to hepatocytes [36], which might explain the findings of our study. Our results for AST and ALT are in accordance with previous reports that associate the modulated activities of AST and ALT after microplastic exposure with tissue damage, inflammation, or the onset and progression of disease due to their impact on intracellular glutamate levels [37,38] and increased prooxidant capacity of cells. Bilirubin is, besides ALT, considered a specific marker of liver injury, and it can be found in high concentrations in hepatocytes [39]. On the other hand, GGT present in hepatocytes and biliary epithelial cells serves as a marker of hepatobiliary injury [40]. GGT is also frequently used to confirm the hepatic origin of increased ALP levels, as this phosphatase mainly originates from two sources—liver and bone [41]. As presented in Table 3, bilirubin, GGT, and ALP exhibited no significant variation across all MP groups compared to Q.

Furthermore, comparing to the Q group, serum analysis revealed unchanged total cholesterol, HDL, and LDL levels in all MP groups, while treatment affected the levels of triglycerides (F_(3, 16)_ = 3.797, *p* = 0.0313), which were elevated in the P2 and P3 groups (*p* = 0.0485 and *p* = 0.0408, respectively) (Table 3). The data in the literature point to the link between the increased levels of triglycerides and perturbed lipid metabolism after MP treatments, due to the gene downregulation of enzymes responsible for the oxidation of fatty acids and disturbed fatty acid metabolism. Moreover, elevated triglycerides in the bloodstream might be responsible for energy production crucial for stress diminution following exposure [37,42,43]. Modulated triglyceride levels are also considered as the serum biomarkers associated with higher coronary risk factors [44], along with increased levels of AST [45]. Thus, further investigation employing additional biomarkers is necessary for a comprehensive understanding of the impact of the MPs on myocardial and liver biliary function.

Our study findings indicate that MP treatments do not affect glucose levels in rats, since similar levels were observed in the serum of all investigated groups (Table 3). The prolonged intake of polystyrene MPs with similar particle size and at a dosage falling within the range of the two higher doses leads to elevated glucose levels and promotes the development of insulin resistance [46]. These effects might be a result of an altered gut microbiota and MP accumulation in various tissues, triggering inflammation and suppression of the insulin signaling pathway [46]. These discrepancies can be attributed to a single MP intake, as well as the regimen of application, and different types of MP usage along with different animal model systems.

Together with liver, the kidneys represent another detoxifying organ, having a vital role in the excretion of waste products and toxins such as urea, creatinine, and uric acid, as well as the regulation of extracellular fluid volume, serum osmolality, and electrolyte concentrations [47]. Urea and creatinine are sensitive biomarkers of kidney function, and when present at abnormal levels, indicate pathological conditions, not only renal disease, but also liver disease, heart failure, or dietary issues [47,48]. Emerging evidence points out that rats chronically exposed to polystyrene nanoparticles and fish exposed to MPs and/or paraquat show elevated creatinine levels, which may indicate kidney toxicity [49,50]. Interestingly, our study also demonstrated that kidneys might be affected by a single MP intake dose (F_(3, 16)_ = 5.717, *p* = 0.0074), as obtained results revealed elevated levels of urea in the two higher MP-treated groups with respect to the Q group (*p* = 0.0467 for P2 and 0.0138 for P3) (Table 3). The presented findings are in accordance with previously published reports indicating that MP exposure could initiate renal nephron injury and modulate the glomerular filtration rate [37,38] as a result of the disruption of the key metabolic pathways crucial for the urea cycle [51]. Considering creatinine, MP treatments affected its level (F_(3, 16)_ = 6.427, *p* = 0.0046) and led to an increase in this parameter in the P2 and P3 groups relative to the Q group (*p* = 0.0339 and 0.0102, respectively) and in P3 compared to the P1 group (*p* = 0.0364) (Table 3), indicating that the kidney function might be compromised. Increased serum creatinine levels might be associated with kidney deterioration due to modulated production and possibly mobilization of creatine and creatinine to meet increased energy requirements as a result of oxidative stress and homeostatic antioxidant scavenging response [52]. More detailed experiments are essential to confirm our results and elucidate the mechanisms by which MPs affect the structural and functional features of different organs.

**Figure 3:**
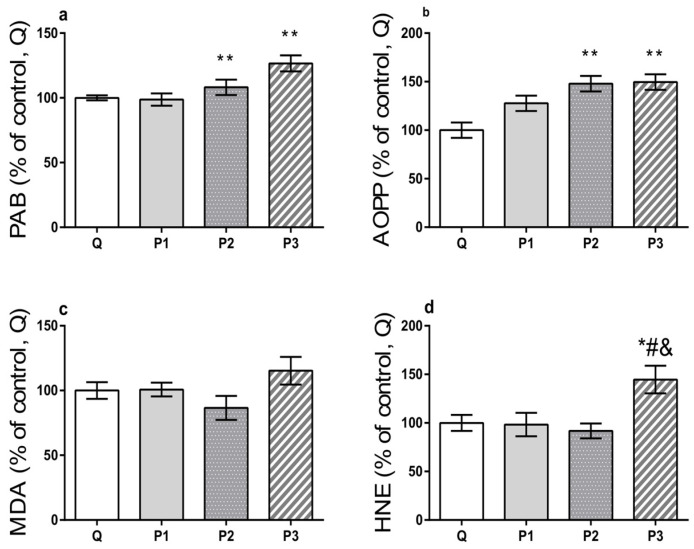
Levels of oxidative stress indicators in serum of adult male Wistar rats 24 h after intragastric treatments. Q group—rats that received 2.5 mL of Milli-Q; P1 group—rats that received 1.4 mg/kg of microplastic particles (MPs) dispersed in 2.5 mL of Milli-Q; P2 group—rats that received 35 mg/kg of MPs dispersed in 2.5 mL of Milli-Q; P3 group—rats that received 125 mg/kg of MPs dispersed in 2.5 mL of Milli-Q. (**a**) Changes in the levels of prooxidant–antioxidant balance (PAB); (**b**) advanced oxidation protein products (AOPP); (**c**) end products of lipid peroxidation: malondialdehyde (MDA) and (**d**) 4-hydroxinonenal (HNE). Data are presented as the mean ± SEM, whereas the values of the Q group were set as 100%. Statistical significance * *p* < 0.05 for P3 vs. Q group; ** *p* < 0.01 for P2 and P3 groups vs. Q group; # *p* < 0.05 for P3 group vs. P1 group and & *p* < 0.05 for P3 group vs. P2 group.

Chronic MP exposure provokes disruptions in energy and lipid metabolism and initiates oxidative stress in rats [3]. Moreover, in human cell cultures, polyethylene particles generate reactive oxygen species (ROS) and increased MDA levels, leading to oxidative stress upon semi-acute and chronic exposure [53]. It remains unknown whether acute treatment with MPs originating from PET is sufficient to modulate oxidative stress parameters, including PAB, AOPP, MDA, and HNE. In our experimental setup, the balance between prooxidants and antioxidants was shifted towards prooxidants (F_(3, 16)_ = 8.488, *p* = 0.0013), particularly in the P2 and P3 groups compared to the Q group (*p* = 0.0097 and 0.0014 respectively) (Figure 3a).

The AOPP, which might be used as an indicator of protein damage promoted by oxidative stress in several pathological conditions like uremia and chronic renal failure [15], was also found to be significantly affected by acute MP treatments (F_(3, 16)_ = 8.413, *p* = 0.0014) and higher in groups treated with higher doses of MPs comparing to the Q group (*p* = 0.0031 for P2 and *p* = 0.0023 for P3) (Figure 3b). Although none of the MP treatments resulted in a statistically significant alteration of MDA (Figure 3c), the HNE levels were affected (F_(3, 16)_ = 4.934, *p* = 0.0130) and increased in the P3 group compared to all other investigated groups (*p* = 0.0472, 0.0380, and 0.0164 for P3 group vs. Q, P1, and P2 groups) (Figure 3d). The observed phenomena, which encompassed serum prooxidant–antioxidant imbalance, protein damage, along with lipid peroxidation, may confirm the generation of oxidative stress caused by excessive production of ROS and/or reduced antioxidative defense in tissues and organs of interest, which could be associated with their disrupted functions provoked by MP pollution.

In summary, this study has demonstrated that a single exposure to MPs has significant impacts on parameters linked to vital organs, notably the heart, liver, and kidneys, with the latest one emerging as a potential focal point. Nonetheless, further studies and evaluations of the short-term effects of MPs are needed to establish unequivocal conclusions.

## 4. Conclusions

Herein, we implemented a novel method for the acquisition of MPs allows the generation of environmentally realistic fragmented fibrous MPs in a relatively inexpensive, simple, and rapid in-house method, and thus holds significant promise for adoption in forthcoming toxicity investigations. Although no changes were observed in the sensorimotor function and clinical signs of toxicity, their slight decrease, accompanied by altered victual consumption and serum biochemical parameters, may indicate that a single MP intake may provoke the development of local changes in tissues and/or organs even after a short period. Despite being considered an inert material, extensive plastic utilization can have detrimental effects on the environment and organisms, including humans, animals, and plants. Therefore, its excessive everyday use should be approached with caution.

## Figures and Tables

**Table 1:** Average food and water intake as indicators of the general health status of adult male Wistar rats 24 h after intragastric treatments. Q group—rats that received 2.5 mL of Milli-Q; P1 group—rats that received 1.4 mg/kg of microplastic particles (MPs) dispersed in 2.5 mL of Milli-Q; P2 group—rats that received 35 mg/kg of MPs dispersed in 2.5 mL of Milli-Q; P3 group—rats that received 125 mg/kg of MPs dispersed in 2.5 mL of Milli-Q. Data are presented as the mean ± SEM. Statistical significance * *p* < 0.05 for P2 and P3 groups vs. Q group.

	Group	Q	P1	P2	P3
Parameter					
Food intake (g)		25.20 ± 0.49	23.80 ± 0.73	20.40 ± 1.47 *	20.60 ± 1.47 *
Water intake (mL)		51.80 ± 0.49	50.60 ± 0.98	45.80 ± 1.71 *	46.00 ± 1.22 *

**Table 2:** Clinical signs of toxicity as indicators of the general health status of adult male Wistar rats 24 h after intragastric treatments. Q group—rats that received 2.5 mL of Milli-Q; P1 group—rats that received 1.4 mg/kg of microplastic particles (MPs) dispersed in 2.5 mL of Milli-Q; P2 group—rats that received 35 mg/kg of MPs dispersed in 2.5 mL of Milli-Q; P3 group—rats that received 125 mg/kg of MPs dispersed in 2.5 mL of Milli-Q. Data are presented descriptively (no effect).

	Group	Q	P1	P2	P3
Parameter					
Agitation		-	-	-	-
Convulsion		-	-	-	-
Ataxia		-	-	-	-
Touch response		-	-	-	-
Piloerection		-	-	-	-
Sleepiness		-	-	-	-
Lethargy		-	-	-	-
Respiratory distress		-	-	-	-
Mortality		0/5	0/5	0/5	0/5

**Table 3:** Levels of markers of organ/tissue function in serum of adult male Wistar rats 24 h after intragastric treatments. Q group—rats that received 2.5 mL of Milli-Q; P1 group—rats that received 1.4 mg/kg of microplastic particles (MPs) dispersed in 2.5 mL of Milli-Q; P2 group—rats that received 35 mg/kg of MPs dispersed in 2.5 mL of Milli-Q; P3 group—rats that received 125 mg/kg of MPs dispersed in 2.5 mL of Milli-Q. Aspartate transaminase (AST), alanine transaminase (ALT), bilirubin, gamma-glutamyl transferase (GGT), alkaline phosphatase (ALP), cholesterol, high-density lipoprotein (HDL), low-density lipoprotein (LDL), triglycerides, glucose, urea, and creatinine. Data are presented as the mean ± SEM. Statistical significance * *p* < 0.05 for P1, P2, and P3 groups vs. Q group and # *p* < 0.05 for P3 group vs. P1 group.

	Group	Q	P1	P2	P3
Parameter					
AST (U/L)		118.00 ± 7.23	131.00 ± 10.25	163.20 ± 9.61 *	167.80 ± 13.84 *
ALT (U/L)		46.00 ± 5.33	24.80 ± 4.68 *	37.6 ± 2.44	40.20 ± 4.33
Bilirubin µmol/L)		0.94 ± 0.07	1.04 ± 0.07	1.08 ± 0.13	1.06 ± 0.07
GGT (U/L)		0.18 ± 0.02	0.18 ± 0.02	0.22 ± 0.02	0.20 ± 0.05
ALP (U/L)		114.40 ± 6.29	119.60 ± 6.81	123.80 ± 6.95	129.20 ± 7.04
Cholesterol (mmol/L)		1.88 ± 0.16	2.08 ± 0.17	2.14 ± 0.13	2.14 ± 0.08
HDL (mmol/L)		1.40 ± 0.11	1.42 ± 0.11	1.44 ± 0.15	1.32 ± 0.13
LDL (mmol/L)		1.30 ± 0.07	1.28 ± 0.09	1.46 ± 0.09	1.48 ± 0.10
Triglycerides (mmol/L)		1.92 ± 0.16	2.36 ± 0.16	2.56 ± 0.12 *	2.58 ± 0.18 *
Glucose (mmol/L)		7.80 ± 0.50	7.94 ± 0.45	8.32 ± 0.53	8.16 ± 0.54
Urea (mmol/L)		8.02 ± 0.57	8.66 ± 0.50	10.66 ± 0.83 *	11.22 ± 0.63 *
Creatinine (µmol/L)		43.80 ± 4.50	47.20 ± 2.96	60.20 ± 4.37 *	63.40 ± 3.01 *#

## Data Availability

Data are contained within the article.

## Supplementary Materials

The following supporting information can be downloaded at: https://www.mdpi.com/article/10.3390/toxics12030167/s1, File Manuscript Supplementary containing: Table S1: Average food and water intake as indicators of the general health status of adult male Wistar rats 24 h after intragastric treatments; Table S2: Sensorimotor functions as indicators of the general health status of adult male Wistar rats 24 h after intragastric treatments; Table S3: Clinical signs of toxicity as indicators of the general health status of adult male Wistar rats 24 h after intragastric treatments; Table S4: Levels of markers of organ/tissue function in serum of adult male Wistar rats 24 h after intragastric treatments; Table S5: Levels of oxidative-stress indicators in serum of adult male Wistar 24 h after intragastric treatments.
