# Supplementary material for: Acute Toxicity Assessment of Orally Administered Microplastic Particles in Adult Male Wistar Rats
Source: Toxics. 2024 Feb 22;12(3):167. doi: 10.3390/toxics12030167 (PMC10974097; doi:10.3390/toxics12030167)
Supplement: Supplementary file 1 [file toxics-12-00167-s001.zip › toxics-2860784-supplementary.pdf]

Supplementary material for

Acute toxicity assessment of orally administered microplastic particles in adult male Wistar rats

Ivana Guševac Stojanović[1*], Dunja Drakulić[1], Ana Todorović[1], Jelena Martinović[1], Nenad Filipović[2], Zoran Stojanović[2*]

[1] Department of Molecular Biology and Endocrinology, VINČA Institute of Nuclear Sciences–National Institute of the Republic of Serbia, University of Belgrade, Mike Petrovića Alasa 12–14, 11351 Belgrade, Serbia; igusevac@vin.bg.ac.rs (I.G.S.), drakulic@vin.bg.ac.rs (D.D.), anato@vin.bg.ac.rs (A.T.), jelenazlatkovic@vin.bg.ac.rs (J.M.)

[2] Group for Biomaterials and Biomedical Applications, Institute of Technical Sciences of SASA, Knez Mihailova Street 35/4, 11000 Belgrade, Serbia; nenad.filipovic@itn.sanu.ac.rs (N.F.), zoran.stojanovic@itn.sanu.ac.rs (Z.S.)

* Correspondence: igusevac@vin.bg.ac.rs (I.G.S.), Tel.: +38117443619; zoran.stojanovic@itn.sanu.ac.rs (Z.S.), Tel.: +38114231952

Funding: This research was funded by the Ministry of Science, Technological Development and Innovation of the Republic of Serbia, grants 451-03-66/2024-03/200017 and 451-03-66/2024-03/200175Acknowledgement: We express our gratitude to Nevena Guševac and Dragan Guševac for their valuable technical support in obtaining microplastic particles.

Conflict of Interest: The authors declare no conflict of interest. The funders had no role in the design of the study; in the collection, analysis, or interpretation of data; in the writing of the manuscript; or in the decision to publish the results

The systematic monitoring (average food and water intake, sensorimotor deficits, clinical signs of toxicity, and levels of biochemical parameters) was performed to detect potential alterations between intact (I) and Q (Milli-Q treated) rats during animals' life and at the terminal experimental endpoint. The rats in I group were not subjected to any specific treatment, while animals in Q group received 2.5 ml of Milli-Q by oral gavage. As presented in **Tables S1 – S5**, no variations in all investigated parameters between these experimental groups were observed, so the intact animals were excluded from further consideration whereas the rats in the Q group served as controls.

Table S1. Average food and water intake as indicators of the general health status of adult male Wistar rats 24 h after intragastric treatments. I group–intact rats; Q group–rats that received 2.5 ml of Milli-Q. Data are presented as the mean ± SEM.

Groups / Parameters	I	Q
Food intake (g)	23.80 ± 0.73	25.20 ± 0.49
Water intake (ml)	53.80 ± 0.73	51.80 ± 0.49

Table S2. Sensorimotor functions as indicators of the general health status of adult male Wistar rats 24 h after intragastric treatments. I group–intact rats; Q group–rats that received 2.5 ml of Milli-Q. Data are presented as the mean ± SEM whereas the values of the I group were set as 100%.

Groups / Parameter	I	Q
Sensorimotor functions	100 ± 1.12	98.90 ± 1.38

Table S3. Clinical signs of toxicity as indicators of the general health status of adult male Wistar rats 24 h after intragastric treatments. I group–intact rats; Q group–rats that received 2.5 ml of Milli-Q. Data are presented descriptively (−no effect).

Groups / Parameters	I	Q
Agitation	-	-
Convulsion	-	-
Ataxia	-	-
Touch response	-	-
Piloerection	-	-
Sleepiness	-	-
Lethargy	-	-
Respiratory distress	-	-
Mortality	0/5	0/5

Table S4. Levels of markers of organ/tissue function in serum of adult male Wistar rats 24 h after intragastric treatments. I group–intact rats; Q group–rats that received 2.5 ml of Milli-Q. Aspartate transaminase (AST), alanine transaminase (ALT), bilirubin, gamma-glutamyl transferase (GGT), alkaline phosphatase (ALP), cholesterol, high density lipoprotein (HDL), low density lipoprotein (LDL), triglycerides, glucose, urea and creatinine. Data are presented as the mean ± SEM.

Groups / Parameters	I	Q
AST (U/L)	129.80 ± 8.06	118.00± 7.23
ALT (U/L)	52.80 ± 3.94	46.00 ± 5.33
Bilirubin (μmol/L)	0.84 ± 0.05	0.94 ± 0.07
GGT (U/L)	0.16 ± 0.02	0.18 ± 0.02
ALP (U/L)	113.00 ± 8.00	114.40 ± 6.29
Cholesterol (mmol/L)	1.84 ± 0.12	1.88 ± 0.16
HDL (mmol/L)	1.42 ± 0.10	1.40 ± 0.11
LDL (mmol/L)	1.22 ± 0.10	1.30 ± 0.07
Triglycerides (mmol/L)	1.92 ± 0.18	1.92 ± 0.16
Glucose (mmol/L)	7.86 ± 0.42	7.80 ± 0.50
Urea (mmol/L)	8.00 ± 0.57	8.00 ± 0.57
Creatinine (μmol/L)	41.40 ± 3.30	43.80 ± 4.50

Table S5. Levels of oxidative-stress indicators in serum of adult male Wistar 24 h after intragastric treatments. I group–intact rats; Q group–rats that received 2.5 ml of Milli-Q. Prooxidant-antioxidant balance (PAB), advanced oxidation protein products (AOPP), end products of lipid peroxidation: malondialdehyde (MDA) and 4-hydroxinonenal (HNE). Data are presented as the mean ± SEM, whereas the values of the I group were set as 100%.

Groups / Parameters	I	Q
PAB (HKU)	100 ± 0.56	95.77 ± 1.84
AOPP (μmol/l)	100 ± 7.64	100.07 ± 7.95
MDA (μM)	100 ± 3.87	97.60 ± 3.74
HNE (μM)	100 ± 12.80	111.84 ± 9.15
